# Effects of Transcranial Electrical Stimulation on Gambling and Gaming: A Systematic Review of Studies on Healthy Controls, Participants with Gambling/Gaming Disorder, and Substance Use Disorder

**DOI:** 10.3390/jcm12103407

**Published:** 2023-05-11

**Authors:** Marija Stanković, Jovana Bjekić, Saša R. Filipović

**Affiliations:** Human Neuroscience Group, Institute for Medical Research, University of Belgrade, 11000 Belgrade, Serbia; jovana.bjekic@imi.bg.ac.rs (J.B.); sasa.filipovic@imi.bg.ac.rs (S.R.F.)

**Keywords:** transcranial electrical stimulation (tES), transcranial direct current stimulation (tDCS), transcranial alternating current stimulation (tACS), gambling, gaming, risk taking, systematic review, cognitive tasks

## Abstract

Gambling disorder (GD) and internet gaming disorder (IGD) are formally recognized behavioral addictions with a rapidly growing prevalence and limited treatment options. Recently, transcranial electrical stimulation (tES) techniques have emerged as potentially promising interventions for improving treatment outcomes by ameliorating cognitive functions implicated in addictive behaviors. To systematize the current state of evidence and better understand whether and how tES can influence gambling and gaming-related cognitive processes, we conducted a PRISMA-guided systematic review of the literature, focusing on tES effects on gaming and gambling in a diverse range of population samples, including healthy participants, participants with GD and IGD, as well as participants with substance abuse addictions. Following the literature search in three bibliographic databases (PubMed, Web of Science, and Scopus), 40 publications were included in this review, with 26 conducted on healthy participants, 6 focusing on GD and IGD patients, and 8 including participants with other addictions. Most of the studies targeted the dorsolateral prefrontal cortex, using transcranial direct current stimulation (tDCS), and assessed the effects on cognition, using gaming and gambling computerized cognitive tasks measuring risk taking and decision making, e.g., balloon analogue risk task, Iowa gambling task, Cambridge gambling task, etc. The results indicated that tES could change gambling and gaming task performances and positively influence GD and IGD symptoms, with 70% of studies showing neuromodulatory effects. However, the results varied considerably depending on the stimulation parameters, sample characteristics, as well as outcome measures used. We discuss the sources of this variability and provide further directions for the use of tES in the context of GD and IGD treatment.

## 1. Introduction

Gambling and gaming are reward-based activities that many people engage in for fun and leisure. However, it has long been known that excessive engagement in certain rewarding behaviors may lead to addiction-like symptoms, often grouped under the term non-substance or behavioral addictions (BAs). Gambling and gaming represent behaviors considered to be addictive, as attested by the ever-rising prevalence of problematic gaming and gambling across the world [1]. This is why gambling disorder (GD) and internet gaming disorder (IGD) have recently been added to the International Classification of Diseases, 11th edition (ICD-11), as formal diagnoses of BAs alongside substance use disorders (SUDs) [2]. The reclassification in ICD-11 has made GD and IGD the first and only two officially recognized BAs. They are defined as patterns of gambling/gaming behaviors characterized by loss of control over the activity, prioritizing gambling/gaming over other activities, and continuation of gambling/gaming despite the negative consequences [2].

Gambling and gaming share common mechanisms that keep people engaged. The aesthetical and functional components of gambling and video games activate dopaminergic reward circuitry, which also plays a significant role in SUDs [3,4]. The similarities between gambling and gaming are reflected in the large overlap between the definition and symptomatology of GD and IGD. At the neural level, individuals with GD and IGD show alterations in fronto-striatal and prefrontal brain regions [3,4,5,6]. Accordingly, studies show that GD and IGD affect the same cognitive functions, including decision making, cognitive control, and reward sensitivity [4,7,8]. In addition, typical cognitive distortions responsible for the maintenance of GD (e.g., near-miss effect and loss-chasing behaviors) can be present during disordered gaming [9,10]. Thus, recent findings about the co-occurrence of problematic gambling and gaming among youth do not come as a surprise [11].

The overlap between gambling and gaming is nowadays even more pronounced, with the expansion of online games offering options to pay for additional features. Furthermore, the Internet has brought significant changes to gambling patterns, with many online gambling activities resembling video games. This rapid growth of the gambling and gaming industries has led to an increased number of people demonstrating symptoms of GD or IGD [1]. While the prevalence of both disorders continues to grow, there is a need for more efforts to develop effective treatment options.

Recently, transcranial electrical stimulation (tES) has emerged as a promising tool for the complementary treatment of various psychiatric disorders, including addiction disorders [12,13]. TES techniques are safe, inexpensive, painless, and easy-to-use forms of non-invasive brain stimulation (NIBS), which makes them particularly appealing [14,15,16]. During tES, a weak electrical field is formed between two or more electrodes placed on the person’s head [17]. The induced electrical field causes local changes in neuronal membrane potentials, bringing neurons closer or further from their firing thresholds [17]. The modulation of neuronal firing patterns can influence cognitive functions controlled by the brain networks targeted with tES [16]. There are different types of tES, depending on the electrode montage and the current waveform that is delivered: transcranial direct current stimulation (tDCS), high definition tDCS (HD-tDCS), transcranial alternating current stimulation (tACS), etc.

Research on healthy participants has shown that tES can influence cognitive processes affected in GD and IGD, such as risky decision making, impulsivity, and reward sensitivity [18,19,20,21]. Furthermore, recent reviews suggest that NIBS techniques can improve clinically relevant outcomes in SUDs by ameliorating cognitive functions and reducing cravings and relapse rates [12,22,23]. These findings have led to the growing popularity of exploring the possibilities of tES in the treatment of BAs. However, due to the large heterogeneity in this field and the small number of studies, reviews on the usage of tES in BAs remain inconclusive [24,25].

Here, we aimed to fill the gap in the existing literature by systematically reviewing tES studies that focused on cognitive functions underlying gambling and gaming behaviors relevant to the GD and IGD development and maintenance. More specifically, we focused on gambling and gaming either in real life or in a laboratory setting measured by cognitive tasks tapping decision making, impulsivity, and/or risk taking (e.g., Iowa gambling task, balloon analogue risk task, etc.).

To fully grasp the scope of tES possibilities for the modulation of gambling/gaming cognitive processes, we first reviewed studies on healthy participants, where tES effects on gaming and gambling tasks were assessed. The tES has been widely used to modulate risk taking or risky decision making in healthy participants, which are the cognitive functions with a pivotal role in GD and IGD development and maintenance. Thus, the first step towards establishing tES as a treatment tool for GD or IGD would be to estimate whether this type of intervention could affect cognitive processes underlying gambling and gaming. Next, we included studies on participants with GD and IGD to review tES effects on task-based measures of gambling and gaming, as well as real clinical outcomes in this population. Finally, we included studies with SUD participants that had gaming and gambling tasks among their tES outcome measures. One of the reasons for this decision is the high comorbidity between behavioral and substance addictions [26]. But more importantly, the suboptimal cognitive functioning leading to increased risk taking and disadvantageous decision making is present in both GD/IGD [7,27,28] and SUDs [29]. In this regard, increased risk taking in GD and IGD populations can be considered a substantial part of the addictive behavior itself, regardless of the likelihood that people with SUDs may develop some of these cognitive deficits as a result of the pharmacological effect of chronic substance use [30]. Thus, by conducting a systematic review of papers that focused on the application of tES for the modulation of cognitive processes relevant to gambling or gaming behaviors in a diverse range of population samples, ranging from healthy participants to participants with GD and IGD and including people with SUDs, we will be able to better understand whether and how tES can affect gambling or gaming-related decision-making processes and whether it can be considered as a promising method to be used in the treatment of GD or IGD.

## 2. Materials and Methods

The review was conducted and reported following the guidelines from the 2020 Preferred Reporting Items for Systematic Review (PRISMA) [31] and has been registered in the PROSPERO International Prospective Register of Systematic Reviews (ID: CRD42023389840).

### 2.1. Eligibility Criteria

We adopted the PICO (participant, intervention, comparator, and outcome) model to specify inclusion and exclusion criteria for the review (see Table 1).

The population of interest was adult human participants, either healthy or presenting with either addictive behavior (subclinical or diagnosed) or an SUD. We included studies that used any form of tES as an intervention tool, either tDCS or tACS. We defined the outcome of interest as performance on cognitive tasks that involve gambling or gaming-related behaviors. Namely, we focused on cognitive tasks that required the same or similar decision-making processes as gambling or gaming, i.e., reward-based decision making, with a certain degree of risk taking involved. As for the studies that included participants with GD or IGD, in addition to the cognitive task performance, we also looked at clinically relevant or behavioral outcomes. More specifically, we opted for investigating the outcomes that can be linked to GD/IGD symptom severity and gambling or gaming propensity, whether they were self-report measures (e.g., self-report craving levels, questionnaires on addictive behaviors) or objective indexes of cognitive/affective functioning (e.g., cognitive tasks measuring cue reactivity, relapse levels, etc.).

The search focused on articles published in peer-reviewed journals in the English language. We included studies that implemented either parallel group or crossover designs with adequate control conditions, i.e., a sham condition against which the tES effects were assessed. Sham treatment refers to delivering a small dose of current for a short time (e.g., 30 s) only at the beginning and sometimes at the end of the session. This is carried out to mimic the sensations of itching/tingling that participants feel during the real session; a small dose of administered current does not produce lasting neurophysiological effects.

### 2.2. Search Strategy and Selection Process

The records for initial screening were identified by a comprehensive literature search conducted in three bibliographic databases (PubMed, Scopus, and Web of Science). The initial search was performed in PubMed and the search string was adjusted for the remaining two databases. Our search strategy combined MeSH terms and keywords in the title and abstract fields that contained: (1) keywords or MeSH terms for tES, such as “transcranial direct current stimulation”, “tDCS”, “tACS”, etc.; (2) keywords or MeSH terms that were relevant to gambling and gaming disorders and tasks, such as “gambl*”, “video game*”, “risk taking”, etc. Exact search strings for each database and information on filters applied can be found in Supplementary Materials. To identify additional relevant publications, the authors manually searched references of the previously identified publications, as well as Google Scholar records, using the “cited by” function.

Final searches were run on 4 January 2023. The study selection was facilitated by Zotero [32] and Rayyan [33] automation tools. M.S. conducted de-duplication in Zotero and the initial screening of the records retrieved from the final search to identify all potentially eligible records and to exclude all irrelevant records, such as review articles and studies conducted on animals. To ensure adequate selection, 13.4% of records were screened by an additional researcher. We retrieved full-text articles of all records marked with ‘’include’’ and ‘’maybe include” tags on Rayyan. Then, two reviewers (M.S. and J.B.) independently assessed whether the selected reports were eligible for the study; if not, they noted the reason for the exclusion. Any conflicting assessments were resolved by all authors.

### 2.3. Data Collection and Synthesis

Upon final selection of studies for inclusion in the review, the following data were extracted from each article: participant population (i.e., sample type: healthy or with a disorder due to substance use or addictive behavior), sample characteristics (size, gender composition), tES modality, stimulation intensity, electrode placement, duration of the stimulation, study design, the study aims, investigated cognitive/emotional function or behavior, cognitive task(s) and outcome measures, and study findings as reported in the results section of the article. One reviewer (M.S.) extracted the data from the papers included and the second author (J.B.) checked and revised the information. We then grouped the studies according to the sample type and separately analyzed the outcomes for healthy and clinical populations. Additionally, we analyzed whether sources of heterogeneity across studies could arise from the extracted data.

### 2.4. Risk of Bias and Quality Assessment

We used the Cochrane risk-of-bias tool for randomized trials (RoB 2) [34] to address the potential issue of bias in different aspects of research reported in selected papers. This tool allows for a structured assessment of potential bias present in five domains, including bias arising from the randomization process, bias due to deviations from intended interventions, bias due to missing outcome data, bias in the measurement of the outcome, and bias in the selection of the reported results.

## 3. Results

### 3.1. Study Selection

**Figure 1:**
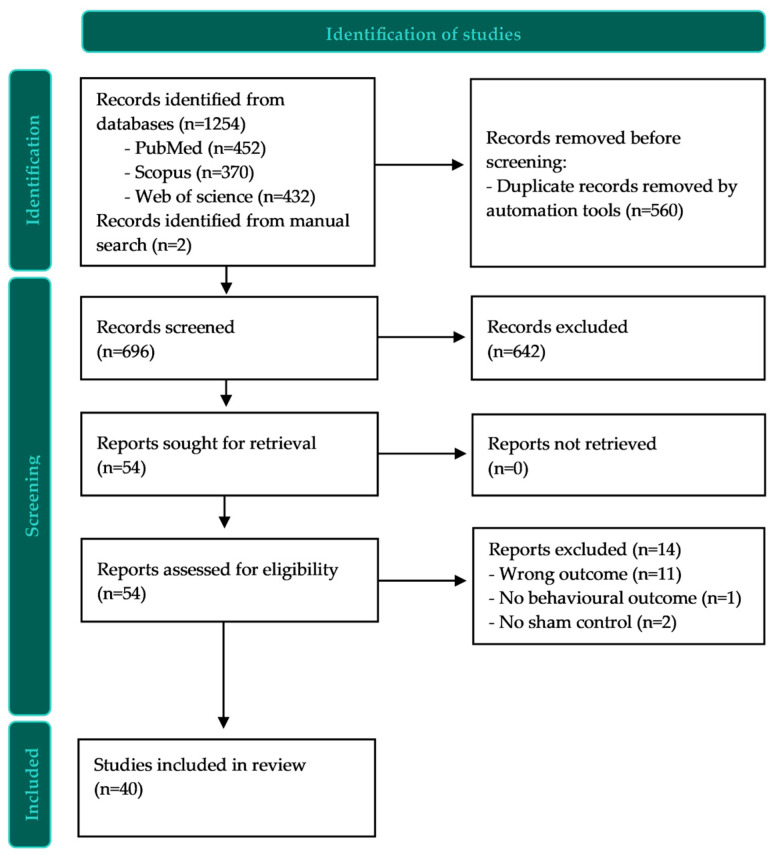
PRISMA flow diagram.

The search resulted in 696 studies for screening after de-duplication. Figure 1 shows the flow of the study selection process. The study selection resulted in a total of 40 studies that met the inclusion criteria.

### 3.2. Study Characteristics

Thirty (75%) studies used parallel group designs; the rest applied cross-over designs. Twenty-six studies were conducted on healthy participants, six on participants with GD or IGD, and eight on participants with SUDs.

The most common tES modality applied was a conventional two-electrode tDCS, with 30 (75%) studies reporting using it. The remaining six studies applied tACS and four applied multifocal tDCS (such as high-definition HD-tDCS). Most of the studies (90%) targeted the dorsolateral prefrontal cortex (DLPFC) and the most common montage was bilateral (19 studies). In tDCS studies, the anode was placed over the left DLPFC and the cathode was placed over the right DLPFC (three studies), opposite anode/cathode direction (seven studies), and both variants (nine studies). In addition, nine studies opted for unilateral DLPFC stimulation (with the return electrode often placed on the other cephalic or extracephalic location, e.g., Pz, supraorbital area, deltoid muscle), while five studies compared the effects of bilateral vs. unilateral montage and thus used both. Other stimulation targets included the orbitofrontal cortex (OFC), anterior cingulate cortex (ACC), and posterior cingulate cortex (PCC).

As for the stimulation dose, most studies used common tDCS/tACS intensities of either 1 mA (7 studies), 1.5 mA (11 studies), or 2 mA (20 studies). In addition, one tDCS study used 0.45 mA and one multifocal tDCS used a cumulative intensity of 3 mA. The offline protocol was implemented in 20 studies, while 19 studies investigated online effects of tES. One study investigated both online and offline tES effects, depending on the task used. The details on the stimulation procedures for each study can be found in Table 2, Table 3 and Table 4.

Behavioral outcomes used to evaluate gambling or gaming-related cognitive functions in healthy participants and participants with SUDs included lab-based measures of risk taking/decision making that could be roughly divided into three groups: (1) a task measure of risk taking where participants earn points/money by inflating the balloon that can explode at any point (i.e., balloon analog risk task (BART)); (2) tasks tapping decision making with components resembling real-life gambling and gaming activities such as dices, cards, or slot machines (i.e., Iowa gambling task (IGT), Cambridge gambling task (CGT), game of dice task (GDT), Columbia card task (CCT), etc.); (3) simple choice-based gambling tasks that present participants with either safe or risky options to choose to gain or lose points/money, considering given probabilities to win or lose (e.g., risk measurement table). In addition to cognitive tasks, the studies on GD and IGD populations included questionnaires designed to measure craving, addiction severity levels, and time spent on gambling/gaming.

### 3.3. The tES Effects on Gambling and Gaming-Related Cognitive Tasks in Healthy Participants

Table 2 summarizes the characteristics and main results of studies that used tES to modulate gambling or gaming-related cognitive tasks in healthy participants.

Overall, most studies showed that tES can affect gambling and gaming-related cognitive tasks in healthy participants. However, there was a large variability in the results when it came to the direction of the effects. Studies that used bipolar or multifocal tDCS, for instance, showed that modulation frontal activity could lead to more advantageous decision making and reduction of risk taking behaviors during tasks that involved gambling/gaming [35,36,37,38,39,40,41,42]. On the other hand, in certain studies that used the same or similar electrode configuration, tDCS disrupted decision making and increased risk taking in these tasks [43,44,45]. These opposing effects were observed even within the same study, that is, some of the studies reported both increases as well as decreases in risk taking, depending on the outcome measures or participants’ characteristics [46,47,48,49]. Finally, several studies showed no significant effect of tDCS on gambling and gaming task performance [50,51,52,53]. There was no indication that the variability of the effects could be attributed to differences in either stimulation intensity, tDCS set-up (including the stimulation side), or protocol (offline vs. online).

Only six studies explored the effects of tACS in healthy participants; they all used online stimulation design, i.e., the cognitive tasks were performed during stimulation. The results of the studies that used tACS delivered in the theta frequency gave a rather unclear pattern. Dantas et al. showed that theta-tACS applied over the left DLPFC reduced risk taking in a gambling task [39], whereas Sela et al. [54] showed that during theta-tACS applied over the left DLPFC, participants took more risk when performing the BART task, while Wischnewski et al. [55] found no effects of theta-tACS applied over the DLPFC bilaterally on a gambling task performance (although there was an increase in decision time). Similarly, tACS applied in the beta frequency range over the left DLPFC led to an increase in risky decision making [56], while an improvement of reversal learning in choice-based gambling tasks was seen when it was applied bilaterally over the DLPFC [57].

**Table 2:** Characteristics and main results of studies that used tES techniques to modulate gambling or gaming behaviors in healthy participants.

Study	Sample (Gender) (no. per Group)	Design	Electrode Placement (Anode(s)/Cathode(s) for tDCS); Hz (for tACS)	Protocol/ Intensity/ Duration	Task	Outcome Measures	Main Results
				tDCS			
Cheng et al. [46]	16 (6 males)	Cross-over, single-blind	S1: right DLPFC/left DLPFC S2: left DLPFC/right DLPFC S3: sham	Online, 2 mA, ≈19 min	BART, RGT	BART: average adjusted pumps; the number of exploded balloons. RGT: frequency of choosing safe option; reaction time.	BART: no effects of tDCS on task performance. RGT: During anodal left DLPFC/cathodal right DLPFC tDCS, participants chose fewer risky options. More impulsive individuals showed a more pronounced effect.
Fecteau et al. [37]	47 (11 males) (10 + 10 + 6+ 6 + 10 + 5)	Parallel group, double-blind	G1: right DLPFC/left DLPFC G2: left DLPFC/right DLPFC G3: right DLPFC/SO G4: left DLPFC/SO G5: sham G6: baseline	Online, 2 mA, 15 min	BART	Average adjusted pumps; total earnings.	Both active groups receiving bilateral prefrontal tDCS showed less risk taking compared with participants receiving sham or no stimulation. Unilateral prefrontal tDCS did not affect risk-taking behavior.
Weber et al. [51]	22 (13 males) (11 + 11)	Parallel group, single-blind	G1: right DLPFC/left DLPFC G2: left DLPFC/right DLPFC G3: sham	Offline, 1.5 mA, 15 min	BART	The total number of balloons, the total number of wins, the percentage of wins, mean pumps per balloon, average adjusted pumps, and total earnings.	No behavioral effects of tDCS on BART performance; however, tDCS affected task-related brain connectivity.
Ouellet et al. [38]	45 (16 males) (15 + 15 + 15)	Parallel group, single-blind	G1: right OFC/left OFC G2: left OFC/right OFC G3: sham	Offline, 1.5 mA, 15 min	BART, IGT	BART: average adjusted pumps. IGT: net score.	BART: a trend-level effect of the intervention was observed. IGT: both active groups receiving bilateral OFC tDCS showed more advantageous decision making.
Russo et al., (Study 1) [52]	117 (49 males) (41 + 43 + 33)	Parallel group, double-blind	G1: right DLPFC/left DLPFC G2: left DLPFC/right DLPFC G3: sham	Online, 2 mA, 15 min	BART	Average adjusted pumps; total money earned.	Participants in the right anodal/left cathodal tDCS of the DLPFC exhibited higher risk taking than the participants in the left anodal/right cathodal tDCS. They had a comparable performance with participants from the sham group.
Russo et al., (Study 2a) [52]	48 (16 males) (16 + 16 + 16)	Parallel group, double-blind	G1: right DLPFC/left DLPFC G2: left DLPFC/right DLPFC G3: sham	Online, 2 mA, 15 min	BART	Average adjusted pumps; total earnings	No effects of tDCS on BART performance.
Russo et al., (Study 2b) [52]	33 (14 males) (11 + 11 + 11)	Parallel group, double-blind	G1: right DLPFC/SO G2: left DLPFC/SO G3: sham	Online, 2 mA, 15 min	BART	Average adjusted pumps; total earnings	No effects of tDCS on BART performance.
Nejati et al. [40]	24 (24 males)	Cross-over, single-blind	S1: right DLPFC/left OFC S2: left DLPFC/right OFC S3: sham	Online, 1.5 mA, 15 min	BART	Average adjusted pumps; the overall pumps; total earnings	Both active stimulation conditions led to reduced risk taking during BART. The effect was observed on all outcome variables.
Boggio et al. [43]	28 (3 males) (10 + 9 + 9)	Parallel group, double-blind	G1: right DLPFC/left DLPFC G1: left DLPFC/right DLPFC G3: sham	Online, 2 mA, 15 min	CGT	Percentage of safe options chosen; reaction time	Anodal tDCS of left DLPFC/cathodal right DLPFC increased risk taking in older adults.
Fecteau et al. [36]	36 (11 males) (12 + 12 + 12)	Parallel group, double-blind	G1: right DLPFC/left DLPFC G2: left DLPFC/right DLPFC G3: sham	Online, 2 mA, 15 min	CGT	Percentage of safe options chosen; reaction time	Anodal tDCS of right DLPFC/cathodal left DLPFC decreased risk taking behavior during CGT.
Leon et al. [49]	61 (27 males) (31 + 30)	Parallel group, blinding not reported	G1: right DLPFC/left trapezium G2: sham	Offline, 1.5 mA, 20 min	IGT	Net score	Sex-dependent tDCS effect/active right OFC tDCS increased net scores in women, but in men no effects were found. Lower performers exhibited the largest effect.
Minati et al. [53]	47 (0 males) (15 + 16 + 16)	Parallel group, double-blind	G1: right DLPFC/left DLPFC G2: left DLPFC/right DLPFC G3: sham	Online, 2 mA, 20 min	A gambling task consisting of probability-based choices	The proportion of positive expectation values chosen/negative expectation values rejected; acceptance of gambles, confidence, the amount earned.	tDCS did not affect task performance. Confidence in the responses was affected; participants under right anodal/left cathodal tDCS were more confident in their responses.
Yang et al. [58]	72 (42 males) (12 + 12 + 12 + 12 + 12 + 12)	Parallel group, blinding not reported	G1: right OFC/left OFC G2: left OFC/right OFC G3: right DLPFC/left DLPFC G4: left DLPFC/right DLPFC G5: sham (DLPFC) G6: sham (OFC)	Offline, 2 mA, 20 min	A gambling task measuring risk/ambiguity aversion and preference	No. of constant payoff selection. Estimated parameters for the corresponding preferences of participants were calculated by a utility function.	tDCS did not affect risk aversion. Risk preference decreased following anodal left DLPFC/cathodal right DLPFC stimulation. Ambiguity preference decreased following anodal right OFC/cathodal left OFC stimulation but increased after reversed montage.
Ye et al. [47]	60 (25 males) (20 + 20 + 20)	Parallel group, blinding not reported	G1: right DLPFC/left DLPFC G2: left DLPFC/right DLPFC G3: sham	Offline, 2 mA, 15 min	RMT (gain-framed and loss-framed choices)	Weighted risk aversion	Anodal right DLPFC/cathodal left DLPFC tDCS resulted in increased risk taking in gain frames and reduced risk taking in loss frames.
Ye et al. [45]	60 (24 males) (20 + 20 + 20)	Parallel group, blinding not reported	G1: right DLPFC/left DLPFC G2: left DLPFC/right DLPFC G3: sham	Offline, 2 mA, 15 min	RMT	Number of safe options chosen	Both groups receiving active stimulation increased risk-taking behavior compared with sham.
Huang et al. [48]	150 (68 males) (30 + 30 + 30 + 30 + 30)	Parallel group, blinding not reported	G1: left DLPFC/Pz G2: right DLPFC/Pz G3: Pz/left DLPFC G4: Pz/right DLPFC G5: sham	Offline, 2 mA, 15 min	RMT	Crossover points: a point when participants switch from safe option toward risky option	Anodal left DLPFC tDCS led to reduced risk taking in the gain-framed form of RMT. Cathodal right DLPFC tDCS led to increased risk taking in the loss-framed form of RMT.
Ye et al. [59]	100 (36 males) (20 + 20 + 20 + 20 + 20)	Parallel group, single-blind	G1: left DLPFC/Pz G2: right DLPFC/Pz G3: Pz/left DLPFC G4: Pz/right DLPFC G5: sham	Offline, 2 mA, ≈20 min	RMT	Number of safe options chosen	Anodal right DLPFC tDCS decreased risk taking.
Xiong, et al. [60]	90 (50 males) (29 + 31 + 30)	Parallel group, single-blind	G1: right DLPFC/left deltoid muscle G2: left deltoid muscle/right DLPFC. G3: sham	Offline, 1.5 mA, 20 min	Gambling tasks measuring risk/ambiguity aversion and preference	Certainty equivalent: the amount of money that caused participants to switch to choosing safe options	Anodal stimulation of the right DLPFC led to a greater preference for ambiguity.
tACS							
Wischnewski et al. [50]	18 (3 males)	Cross-over, double blind	S1: AF3/FC1; AF4/FC2; 5 Hz S2: AF3/AF4; FC1/FC2; 5 Hz S3: Sham	Online, 1 mA peak-to-peak 30 min	GDT	Estimation of behavioral pattern, stop probability, exploration factor; uncertainty value (RT-based measure)	No tACS effects on measures of risk taking, stop probability and exploration. During both active TACS conditions, participants were more uncertain in their choices.
Dantas et al. [39]	32 (16 males)	Cross-over, single-blind	S1: left DLPFC/circular electrode around it; 6.5 Hz S2: left DLPFC/circular electrode around it; 40 Hz S3: sham	Online, 1.5 mA, peak-to-peak 30 min	CGT	Computed level of risk, choice of probability, the average value chosen, response time	Theta tACS significantly reduced risk-taking behavior compared with sham and gamma tACS.
Sela et al. [54]	27 (13 males) (9 + 8 + 10)	Parallel group, double-blind	G1: left DLPFC/right temporal lobe; 6.5 Hz G2: right DLPFC/left temporal lobe; 6.5 Hz G3: sham	Online, 1 mA peak-to-peak, 15 min	BART	Average adjusted pumps; number of exploded balloons	Left DLPFC theta tACS increased risk-taking behavior; the participants made more pumps on the BART and had a larger number of balloon explosions.
Yaple et al. [56]	34 (13 males) (17 + 17)	Parallel group, blinding not reported	G1: left DLPFC/left deltoid muscle; 5 Hz, 10 Hz, 20 Hz, 40 Hz G2: right DLPFC/right deltoid muscle; 5 Hz, 10 Hz, 20 Hz, 40 Hz G3: sham	Online, 1 mA peak-to-peak, 5–10 min	Choice-based gambling task	Selection of risky decisions and selection of switches between tasks	Increased risky decision making during 20 Hz tACS applied over left DLPFC compared with sham. Other tACS frequencies showed no significant effect.
Wischnewski et al. [57]	108 (35 males) (36 + 36 + 36)	Parallel group, double-blind	G1: AF3/FC1; AF4/FC2; 20 Hz G2: AF3/AF4; FC1/FC2; 20 Hz G3: sham	Online, 1 mA peak-to-peak ≈13 min	Choice-based gambling task (reversal learning task)	Probability for high-risk/low-risk choices across task blocks	Beta tACS improved rule implementation in the reversal learning task; the participants in both active stimulation groups made more advantageous choices compared with the sham group.
Wischnewski et al. [55]	50 (29 males) (25 + 25)	Parallel group, double-blind	G1: left DLPFC/right DLPFC (electrodes were positioned slightly under F3/F4); 6 Hz G2: sham	Online, 1 mA peak-to-peak ≈12 min	Choice-based gambling task (reversal learning task)	Probability for high-risk/low-risk choices across task blocks	Participants receiving theta tACS learned the rules of the task faster than the ones in the sham group. Additionally, they showed a decrease in high risk taking after reversal learning.
Multifocal tDCS							
Mattavelli et al. [41]	20 (10 males)	Cross-over, single-blind	S1: dACC: Fz-F1-FCz/PO9-O9-O10 S2: dACC: PO9-O9-O10/Fz-F1-FCz S3: sham	Offline, 3 mA 20 min	Choice-based gambling tasks (loss and risk aversion tasks)	Loss aversion, risk aversion, choice consistency parameters	Cathodal stimulation of dACC stimulation reduced risk-taking behavior and increased loss aversion.
Guo et al. [35]	58 (21 males) (20 + 16 + 22)	Parallel group, single-blind	G1: left DLPFC/AF3-F1- F5-FC3. G2: AF3-F1-F5-FC3/left DLPFC G3: sham	Online, 1.5 mA, 20 min	BART	Average adjusted pumps, total money earned, number of explosions	Participants in the cathodal HD-tDCS group earned less money in BART compared with participants in the sham group. However, no effect of the stimulation on adjusted pumps or the number of explosions was observed.
He et al. (Study 1) [42]	41 (41 males) (22 + 19)	Parallel group, single-blind	G1: left DLPFC: F1/F5-AF3-FC3 G2: sham	Offline, 1.5 mA 20 min	IGT	Net score	HD-tDCS group learned the rules in IGT faster than the sham group, leading to higher IGT scores.
He et al. (Study 2) [42]	49 (49 males) (23 + 26)	Parallel group, single-blind	G1: right DLPFC: F2/F6-AF4-FC4 G2: sham	Offline, 1.5 mA 20 min	IGT	Net score	No effects of HD-tDCS on the performance IGT task performance.
He et al. (Study 3) [42]	20 (20 males)	Cross-over, single-blind	S1: right DLPFC: F1/F5-AF3-FC3 S2: sham	Offline, 1.5 mA 20 min	IGT	Net score	After HD-tDCS, the participants showed faster learning of the IGT rules.
Wang et al. (Study 1) [44]	34 (34 males) (11 + 11 + 12)	Parallel group, blinding not reported	G1: rACC: Cz- Ex10- C2- FT10- Ex5- FC2-FCz/Fpz-Afz G2: PCC: Fz- TP7- O2- P8- FC6- FC5- O9/Pz-CPz G3: sham (M1)	Online, 2 mA 20 min	IGT	Net score	Cathodal HD-tDCS decreased the IGT score in certain blocks in both active groups.
Wang et al. (Study 2) [44]	34 (34 males) (11 + 11 + 12)	Parallel group, blinding not reported	G1: rACC: Cz- Ex10- C2- FT10- Ex5- FC2- FCz/Fpz-Afz G2: PCC: Fz- TP7- O2- P8- FC6- FC5- O9/Pz-CPz G3: sham (M1)	Online, 2 mA, 20 min	Risk decision task	Total score	No significant effect of HD-tDCS on risk decision tasks was found.

Note: G—group; S—session; tDCS—transcranial direct current stimulation; HD tDCS—high definition tDCS; tACS—transcranial alternating current stimulation; BART—balloon analog risk task; IGT—Iowa gambling task; CGT—Cambridge gambling task; GDT—game of dice task; RMT—risk measurement table; RGT—risky gains task; DLPFC—dorsolateral prefrontal cortex; rACC—rostral anterior cingulate cortex; SO—supraorbital area; PCC—posterior cingulate cortex.

### 3.4. The tES Effects in Studies on GD and IGD

The study characteristics and main findings of tES effects on GD and IGD symptoms are summarized in Table 3. Although all six studies targeted DLPFC using tDCS in the offline type of protocol, there was a high variability in the stimulation characteristics. Bilateral montage was more often used (four studies), but in two studies it was in a left-to-right (anode/cathode) direction, while in another two the direction was opposite. Two studies were conducted on patients with GD, while four focused on IGD.

Soyata et al. [61], using bilateral right-to-left montage found that participants with GD improved decision making in IGT and cognitive flexibility in the Wisconsin card sorting task, following three sessions of bilateral tDCS. Further, Martinotti et al. [62], using the same type of montage, showed that five sessions of tDCS could reduce craving, in a mixed sample of patients with SUDs and GD.

As for the research on IGD, Wu et al. [63,64] found that a single tDCS session targeting the right DLPFC could result in an improvement of inhibitory control over gaming-related cues and could also facilitate the regulation of craving and emotions. Using a bilateral left–right montage, Jeong et al. [65] found that 12 active tDCS sessions may decrease time spent on gaming, as well as improve self-control and reward seeking. Conversely, Lee et al. [66], using the same montage, did not observe significant effects of 10 sessions of tDCS on any outcomes, including craving, cognitive control, the severity of addiction, behavioral activation, and inhibition. It might be of note that the intensity of stimulation in the latter was only 1 mA in comparison to 2 mA in the former.

### 3.5. The tES Effects on Gambling and Gaming-Related Cognitive Tasks in Participants with SUDs

Table 4 shows the summary of characteristics and the results of studies that investigated tES effects on gambling/gaming outcomes in people with SUDs. All studies targeted DLPFC with bilateral tDCS. Four single-session studies focused on the immediate effects on the risk-taking tasks related to gambling/gaming behaviors; three of the studies adopted online protocols, while one studied the aftereffects of the stimulation in an offline protocol. They showed that the direction of tES effects on gambling/gaming-related tasks could depend on the sample type, the outcome measure, and/or the stimulation parameters. While Boggio et al. [67] showed that cannabis users increased risk taking in CGT during bilateral stimulation of DLPFC, regardless of whether the anode was left or right, Patel et al. [68] did not find tDCS effects when the anode was left, using the same montage and almost the same study design. In contrast, Pripfl et al. [69] showed that, during bilateral DLPFC stimulation, in the ‘’cold‘’ version of the CCT, both smokers and non-smokers reduced risk taking when the anode was left (when the anode was right, there was no effect), whereas, in the ‘’hot‘’ version of the same task, only smokers decreased, while non-smokers paradoxically increased risk taking, but only when the anode was right (when the anode was left, there was no effect). Moreover, Gorini et al. [70] showed that both cocaine users and healthy controls decreased risk taking following anodal tDCS over the right DLPFC (measured by BART and GDT), while anodal tDCS over the left DLPFC increased risk taking, but only in cocaine users (measured by GDT).

The studies that applied multiple sessions of tDCS also showed mixed effects, despite using the same current intensity (2 mA) and similar protocols. Gilmore et al. [71] found that 10 sessions of tDCS, when the anode was right, coupled with BART reduced risk taking in CGT in a sample of war veterans demonstrating patterns of SUDs. Alizadehgoradel et al. [72] showed that substance users reduced risk taking in BART after 10 sessions of tDCS, but when the anode was left. On the other hand, Fecteau et al. [73] and Verveer et al. [74] did not find significant effects on gambling/gaming task performance after multiple sessions of right anode tDCS in smokers and cocaine users.

**Table 3:** Characteristics and main results of studies that used TES techniques in GD or IGD. All studies used tDCS.

Study	Sample (no. per Group)	GD/IGD	Design	Electrode Placement (Anode/Cathode)	Protocol/ Intensity/ Duration/ No. of Sessions	Task/Scale	Outcome	Main Results
Soyata et al. [61]	20 (20 males) (10 + 10)	GD	Parallel group, triple blind	G1: right DLPFC/left DLPFC G2: sham	Offline, 2 mA, 20 min, three sessions	IGT, WSCT	IGT: net score WSCT: the number of preservative errors	Active tDCS resulted in more advantageous decision making and better cognitive flexibility in GD patients.
Martinotti et al. [62]	34 (28 males, 4 with GD) (18 + 14)	GD + other SUDs	Parallel group, double-blind	G1: right DLPFC/left DLPFC G2: sham	Offline, 1.5 mA, 20 min, five sessions	Visual analog scale for craving, timeline follow back, BIS-11, HAM-D, HAM-A, Y-MRS	Scores on scales	Significant effects of active tDCS on craving reduction were found. No data about GD patients specifically.
Jeong et al. [65]	26 (15 males) (13 + 13)	IGD	Parallel group, single-blind	G1: left DLPFC/right DLPFC G2: sham	Offline, 2 mA, 30 min, 12 sessions	BIS/BAS, IAT, BSCS time spent on gaming	Scores on scales, average weekly hours spent on gaming	No interaction effects between group (active vs. sham) and time were found. However, active tDCS led to a significant decrease in time spent on gaming, as well as increased self-control.
Wu et al. [63]	33 (33 males)	IGD	Cross-over, double-blind	S1: right DLPFC/left trapezius. S2: sham	Offline, 1 mA, 20 min, single session	Cue-induced craving scale; letter categorization task with gaming-related cues as distractors	Score on a craving scale; interference effects	Active tDCS led to improved inhibitory control over gaming-related cues, but it did not affect cue-induced craving.
Wu et al. [64]	33 (33 males)	IGD	Cross-over, double-blind	S1: right DLPFC/left trapezius. S2: sham	Offline, 1 mA, 20 min, single session	Regulation of craving task. Emotional regulation task.	Scores on craving scales	Active tDCS facilitated the upregulation and downregulation of craving levels during tasks.
Lee et al. [66]	26 (16 males) (14 + 12)	IGD	Parallel group, double-blind	G1: left DLPFC/right DLPFC G2: sham	Offline, 1 mA, 20 min, 10 sessions	IAT, craving scale, BDI, BAI, stop signal task	Scores on scales; the number of errors and proportion of successful stops in stop signal task	No behavioral effects of tDCS were observed.

Note: G—group; S—session; tDCS—transcranial direct current stimulation; IGT—Iowa gambling task; DLPFC—dorsolateral prefrontal cortex; WSCT—Wisconsin card sorting task; BIS—behavioral inhibition scale; BAS—behavioral activation scale; HAM-D—Hamilton depression rating scale; HAM-A—Hamilton anxiety rating scale; Y-MRS—Young mania rating scale; BSCS—brief self-control scale; IAT—internet addiction test.

**Table 4:** Characteristics and main results of studies that used TES techniques to modulate gambling or gaming cognitive tasks in participants with SUDs. All studies used tDCS.

Study	Sample (no. per Group)	SUD Type	Design	Electrode Placement (Anode/Cathode)	Protocol/ Intensity/ Duration/ No. of Sessions	Task	Outcome	Main Results
Patel et al. [68]	27 (16 males) (15 + 12)	Cannabis	Parallel group, double-blind	G1: right DLPFC/left DLPFC G2: sham	Online, 2 mA, 15 min, single session	CGT	Percentage of safe choices across trials; reaction time	No significant effect of bilateral DLPFC tDCS on risk taking was found.
Boggio et al. [67]	25 (15 males) (9 + 8 + 8)	Cannabis	Parallel group, double-blind	G1: right DLPFC/left DLPFC G2: left DLPFC/right DLPFC G3: sham	Online, 2 mA, 15 min, single session	CGT	Percentage of safe choices across trials; reaction time	Both active tDCS groups increased risk-taking behavior.
Pripfl et al. [69]	18 smokers (8 males) + 18 non-smokers (3 males)	Smoking	Cross-over, blinding not reported	S1: right DLPFC/left DLPFC S2: left DLPFC/right DLPFC S3: sham	Online, 0,45 mA, 15 min, single session	CCT, two versions (“hot” and “cold”)	The number of cards chosen	Cold version: anodal left/cathodal right DLPFC tDCS resulted in decreased risk taking compared with sham. Hot version: anodal right/cathodal left tDCS of DLPFC decreased risk taking in smokers and increased it in non-smokers.
Gorini et al. [70]	18 cocaine abusers (10 males) + 18 matched controls	Cocaine	Cross-over, single-blind	S1: right DLPFC/left DLPFC S2: left DLPFC/right DLPFC S3: sham	Offline, 1.5 mA 20 min, single session	BART, GDT	BART: average adjusted pumps GDT: average safe bets	BART: both control subjects and cocaine abusers reduced risk taking after both active tDCS. GDT: anodal right DLPFC/cathodal left DLPFC tDCS led to safer bets in all participants. Anodal left DLPFC/cathodal right DLPFC led to more risk-taking behavior in cocaine abusers.
Fecteau et al. [73]	12 (5 males)	Smoking	Cross-over, double-blind	S1–5: right DLPFC/left DLPFC S5–10: sham	Offline, 2 mA, 30 min, five sessions	CGT (money and cigarettes as rewards)	number of low- vs. high-risk options chosen	No significant effects of tDCS on CGT performance.
Verveer et al. [74]	59 (47 males) (29 + 30)	Cocaine	Parallel group, blinding not reported	G1: right DLPFC/left DLPFC G2: sham	Offline, 2 mA, 13 min x 2 rounds, 10 sessions	Choice-based gambling task	The average number of points won per trial; percentage of high-risk over low-risk options chosen	No significant effects of tDCS on risk taking.
Alizadehgoradel et al. [72]	39 (39 males) (19 + 20)	Methamphetamine	Parallel group, double-blind	G1: left DLPFC/right DLPFC G2: sham	Offline, 2 mA, 20 min, 10 sessions	BART	Average adjusted pumps, the maximum number of pumps	Active tDCS led to a lower adjusted number of pumps.
Gilmore et al. [71]	30 (29 males) (15 + 15)	Alcohol + other substances	Parallel group, single-blind	G1: right DLPFC/left DLPFC G2: sham	Online (BART); offline (CGT), 2 mA, 25 min 10 sessions	BART CGT (primary outcome)	BART: average adjusted pumps CGT: percent of choosing the high-risk option	BART: The participants performed BART during each session and a follow-up. No significant differences in risk taking between the active and sham group were found. CGT: active group showed a significant decrease in risk-taking behavior compared with the sham.

Note: G—group; S—session; tDCS—transcranial direct current stimulation; BART—balloon analog risk task; IGT—Iowa gambling task; CGT—Cambridge gambling task; GDT—game of dice task; CCT—Columbia card task; DLPFC—dorsolateral prefrontal cortex.

### 3.6. Risk of Bias

**Figure 2:**
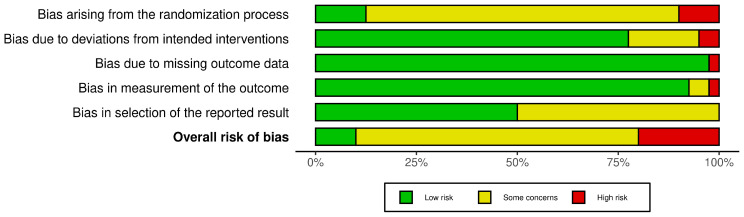
Risk of bias assessment (Robvis visualization tool).

Figure 2 shows the summary result of the risk of bias assessment. The results for each included study can be found in Supplementary Materials. In general, some concerns regarding the risk of bias could be identified in most studies; these were mainly arising from randomization processes, i.e., a lack of adequate description of exact randomization procedures and/or implementation of or reporting on blinding protocols. In addition, the bias of the selection of reported results, mostly stemming from the lack of pre-registration of the statistical analyses, made it difficult to assess whether the reported results were in line with the data analysis plan. In addition, most studies did not report any measure of the successfulness of blinding, thus making it difficult to check the real placebo effect.

## 4. Discussion

The present study represents a comprehensive review of sham-controlled studies that used tES to modulate gambling and gaming-related behaviors. The focus of the review was on tES effects on cognitive processes and behaviors relevant to GD and IGD in healthy participants, participants with GD or IGD, and participants with SUDs. We identified 40 studies matching the inclusion criteria, out of which six focused on GD and IGD participants. Overall, the results indicated that it is possible to affect gambling/gaming-related cognitive processes by using tES. Namely, 70% of studies showed neuromodulatory effects on either gambling/gaming-related cognitive tasks or gambling/gaming behaviors, or both. Reduced risk taking, enhanced decision making, or improvement of GD/IGD symptoms was found in 40% of studies. An additional 20% of the studies showed positive effects that were contingent on either type of outcome measure (e.g., positive effects on one task and null on the other) or person-related characteristics (e.g., baseline personality traits), while the rest showed either effects in the opposite direction (10%) or reported on null findings. However, the effects seemed diverse and dependent on the stimulation parameters, outcome measures used, characteristics of the sample, and methodological aspects of the studies. The following sections focus on examining how these sources of heterogeneity might have contributed to the results of the studies we reviewed here.

### 4.1. Using tES to Modulate Prefrontal Networks Relevant to GD and IGD

Most studies opted for conventional bipolar tDCS, which consists of placing two electrodes of opposite charge (anode and cathode) on the participant’s head to form the electrical field with unidirectional current flow. To modulate GD/IGD functions and behaviors, prefrontal brain areas have been the primary target in tDCS studies. This choice of stimulation site is driven by neuroimaging evidence pointing out DLPFC and OFC as some of the key structures involved in cognitive control [75], as well as in disorders due to substance use or addictive behaviors [76,77]. The idea behind placing electrodes over the prefrontal cortex is that tDCS would improve cognitive control and lead to more favorable decision making. Building up on the hypothesis of an imbalanced activity between the left and right hemispheres in psychiatric disorders affecting decision making [78,79,80], numerous studies decided on the bilateral montage. While the left DLPFC is considered particularly important for regulating negative emotions and controlling impulsive behavior [81], the right DLPFC is thought to be more involved in reward processing and reward-related emotions and motivation [82]. Thus, the imbalance in left/right DLPFC activity may result in cognitive–emotional patterns typically observed in gambling and gaming addiction, i.e., high sensitivity to reward, impulsivity, and impaired cognitive control.

Indeed, many studies found that bi-hemispheric frontal stimulation was associated with the adoption of risk-averse behaviors during gambling/gaming tasks in healthy participants, indicating that balancing the activity across the left and right frontal hemispheres may be crucial for taking control over risky gambling and gaming associated behavior [36,37,38,40]. Moreover, this montage has been shown to be effective in improving cognition and reducing cravings in patients with GD and IGD [61,62,63,64,65], as well as in facilitating decision making, in studies focusing on gambling/gaming behaviors in participants with SUDs [71,72]. Nevertheless, some studies showed null or even the opposite effects [43,53,68,73].

The variable results may highlight the importance of current flow distribution across cortical and subcortical structures and its influence on cognitive functions relevant to gambling/gaming behaviors. Namely, tDCS induces a wide-spread distribution of electrical field in the brain that is not limited to the targeted brain areas only [83]. A recent meta-analysis showed that the density of the current in DLPFC subregions may determine the effects of tDCS, which emphasizes the significance of stimulation focality [84]. To that end, some studies used multifocal tDCS by placing smaller electrodes on multiple points of the scalp, forming a more focal electrical field in the brain [85]. An advantage of multifocal tDCS techniques could lie in their ability to more precisely target other structures important for GD and IGD (e.g., ACC, insula, etc.), which can lead to more robust effects. The studies on healthy participants with optimized electrode montage seem promising [35,41,42,44]; however, this approach is yet to be used in GD and IGD populations.

Additionally, there is evidence that tES effects may be dosage dependent, with higher doses leading to more pronounced effects, at least when it comes to tACS [86]. Even though we did not observe this pattern when analyzing the included studies, dose–response is an important aspect of optimizing tES parameters and it should be further investigated in gambling and gaming-focused tES research, Furthermore, tDCS effects are considered to be brain state dependent [87], which was a factor rarely taken into account in the studies we reviewed here. These aspects may partially explain the different directions of results (e.g., increased vs. decreased risk taking) across the included studies.

Although most studies used a unidirectional constant current, several studies opted for tACS, which delivers a sinusoidally modulated electrical current that switches between polarities in a specific frequency. The technique is used with the aim to entrain endogenous brain oscillatory patterns corresponding with the stimulation frequency [88]. In this review, the tACS studies on risk taking and decision making [39,54,55,56] applied stimulation mostly in theta frequencies, due to the role of 4–8 Hz oscillation in decision making [89] and risk taking [39]. These studies showed promising findings, as theta tACS was able to modulate risk taking [39,54,55]. Still, the frequency-specific effects are uncertain, as two studies showed that beta-band tACS can also modulate reward-based learning during gambling/gaming tasks [57].

Overall, the current state of evidence suggests that targeting DLPFC, either by increasing activity in the left or decreasing activity in the right hemisphere or both in a bilateral electrode montage, can induce changes relevant to gambling and gaming behavior. Optimizing an electrode montage to affect relevant brain structures more focally and to entrain oscillatory activity in relevant brain networks is a promising path to follow, as initial studies show favorable results.

### 4.2. Diversity of Cognitive Functions Relevant for GD and IGD

People who demonstrate problematic gambling and gaming are characterized by impulsiveness, reduced inhibitory control, suboptimal decision making, heightened reward sensitivity, etc. Therefore, in tES studies, GD and IGD-relevant functions can be assessed with a wide range of outcome measures. In this review, we summarized findings from risk-taking tasks, as well as decision-making tasks, with components resembling real-life gambling and gaming activities. The diversity and heterogeneity of the outcome measures (i.e., different types of tasks used in the reviewed studies) may be an important source of the results’ variability. Namely, a conflicting aspect of gambling/gaming tasks is their feature to recruit two distinctive cognitive processes: decision making under risk (when the probability to win is known, e.g., CGT) and decision making under ambiguity (when the probability to win is unknown, e.g., IGT, BART). While both forms of decision making are relevant to GD and IGD, they might engage different neural mechanisms. In fact, several studies found that the same stimulation protocol differently affected performance, depending on the decision-making type required [46,47,48,58]. This is in line with neuroimaging evidence identifying distinct roles of cortical and subcortical regions associated with different types of decision making [90]. For example, the meta-analysis by Krian and colleagues showed that risky decision making relies on OFC and rostral portions of the medial wall, while ambiguous decision making is associated with DLPFC and more caudal portions of ACC [90]. Thus, it is important to be mindful of the type of decision-making task when deciding on electrode positioning i.e., maximizing current intensity in the relevant region of the brain.

### 4.3. Challenges of Studying tES Effects on Gambling and Gaming across Different Populations

In the studies included in this review, gambling- and gaming-related functions were investigated in participants with varying degrees of risk-taking tendencies. Therefore, it was important to take individual differences into account when evaluating tES effects. Some studies showed that tES effects could be trait-dependent, e.g., they found more pronounced tES effects among more impulsive individuals or in females [45,48] and there is evidence that lower baseline performance may lead to better responses to tES [88].

The findings on baseline-dependent effects could be especially relevant for translating basic research results to GD and IGD populations; namely, since people with GD and IGD tend to perform poorer in tasks measuring decision making, cognitive control, and risk taking compared with healthy controls [7], it could be expected that, in them, tES might induce larger and more reliable effects. However, no study directly assessed the differences in responsiveness to tES between GD/IGD and healthy participants and the evidence on the baseline-dependent differences in effect sizes is still insufficient. Clinically oriented studies on gambling and gaming focused more on addiction-related outcome measures (e.g., craving), traits (BIS/BAS), and self-reported or real-life behaviors (e.g., weekly time spent on gaming) than performances in cognitive tasks. Nevertheless, although the number of sham-controlled tES studies in this area is still small and the research is widely heterogeneous in terms of aims, designs, and outcome measures, their results provide solid ground for noting that tES deserves further attention in the research of its therapeutic potential for BAs. It would be advisable that future studies continue investigating tES effects on the mechanism implicated in GD and IGD, focusing on at-risk populations to better determine the optimal stimulation parameters.

Apart from healthy participants and IGD/GD populations, in this review, we included the studies with participants exhibiting various forms of addictive behaviors: using cannabis, cigarettes, cocaine, methamphetamine, and alcohol. We included these studies because of the high comorbidity between different types of addiction [26] and increased risk taking often observed in SUDs; the latter being either a promoter of addictive behaviors or resulting from the pharmacological effect of chronic substance use [30]. The tES studies on gambling and gaming behaviors in SUDs showed mixed findings, which came as no surprise, as SUD participants possibly had specific neurocognitive profiles and certain cognitive deficits stemming from the influence of chemical components of the used substances. Despite these confounding factors, some of the included studies showed group differences in tES effects between healthy and participants with SUDs; depending on the tES protocol, substance users either decreased or increased risk taking more than healthy controls [69,70], which was in line with the notion on trait-dependent tES effects.

### 4.4. Limitations of the Current State of Evidence and Future Directions

The current state of knowledge regarding the tES effects on gambling and gaming is still limited and the studies analyzed here show heterogeneity in the methods as well as the results, making it difficult to draw any firm conclusions.

To move the field further, several methodological aspects that might have contributed to the variable results need to be addressed. First, a significant number of studies were conducted on small samples, making them underpowered and questioning the reliability of the observed findings. Second, the question of proper blinding was often neglected, either by not reporting on blinding at all or adopting a single-blind study design. Furthermore, the successfulness of blinding was rarely assessed, which opens the question whether the observed effects could, at least partially, be attributed to the placebo effect. Third, the majority of studies showed the risk of bias due to the incomplete reporting of procedures (e.g., not reporting on the randomization of participants into groups). Incomplete reporting may impede future replication attempts and meta-analyses. Thus, we encourage authors to report the data as transparently and thoroughly as possible. Preregistration of the planned study protocols and statistical analyses could also be a beneficial practice. Finally, the different methodological choices (e.g., electrode placement, current flow direction, choice of the outcome task, etc.) were rarely presented with a clear rationale. Therefore, it is important that future studies explicitly state which aspects of the work are theory and hypothesis-driven and which are exploratory or a follow-up on accidental findings reported in previous studies.

To assess the effectiveness of tES in the modulation of gaming and gambling-relevant functions, future studies should try to optimize tES delivery to maximize current intensity in the medial frontal lobe structures (e.g., ACC) that play a central role in regulating risky behaviors. This can be achieved by combining tES with neuroimaging methods and current-flow modeling guided-electrode positioning, which could also facilitate finding the optimal dosage for the modulation of relevant brain areas. Furthermore, it is important to establish which cognitive–affective functions/processes that are relevant for gaming and gambling are susceptible to tES-induced neuromodulation. To accomplish this, the outcome measures need to be clearly defined and the stimulation parameters should be tailored to the task-relevant neural activations. Importantly, future studies with GD and IGD populations should systematically follow how specific task performance improvement/disruption is related to the severity of clinical symptoms. Additionally, it is essential to understand the baseline-performance dependence of the stimulation aftereffects as a starting point for the development of tES protocols that can be systematically assessed in at-risk populations and those diagnosed with GD/IGD. Finally, using tES for treatment purposes implies that the effects of the intervention last beyond the duration of the stimulation itself. Even though online protocols can be useful for making causal inferences about the importance of specific brain rhythms and/or brain regions for a cognitive process, it is unclear how these functions are affected after the stimulation. Thus, placing more attention on investigating the strength of offline effects is vital for establishing tES as a therapeutic tool for GD/IGD.

## 5. Conclusions

This systematic review showed that tES can modulate prefrontal networks to induce changes in cognitive functions underlying gambling and gaming behaviors in healthy participants, as well as in people with GD/IGD and other addictions. However, further research is needed to determine the most effective stimulation protocols, as well as to assess the effectiveness of so far not-much-used tES techniques, such as tACS and HD-tDCS/tACS.

## Figures and Tables

**Table 1:** PICO framework-based inclusion and exclusion criteria.

Category	Include	Exclude
Participants	- Adults (>18 years old), healthy or with addictive behavior (gambling disorder (GD) and internet gaming disorder (IGD)) or a substance use disorder (cocaine, alcohol, etc.).	- Non-human participants. - Children. - Human participants suffering from a mental disorder other than addiction.
Intervention	- tES (tDCS or tACS)	- No tES intervention was reported. - Reported additional intervention applied along with tES (e.g., cognitive training).
Comparator	- Sham condition.	- No sham condition.
Outcomes (for studies on healthy participants or participants with SUD)	- Gambling and gaming-related behaviors, as measured by cognitive tasks that involve reward-based decision making and risk taking such as BART, CGT, etc.	- No behavioral outcomes were reported. - Behavioral outcomes that do not include gambling or gaming-related behaviors. - Behavioral tasks that include social decision making (e.g., ultimatum game).
Outcomes (for studies on participants with GD and IGD)	- Any clinically relevant behavioral outcome.	- No behavioral outcomes were reported.

Note: tES—transcranial electrical stimulation; tDCS—transcranial direct current stimulation; tACS—transcranial alternating current stimulation; GD—gambling disorder; IGD—internet gaming disorder; BART—balloon analogue risk task; CGT—Cambridge gambling task.

## Data Availability

No new data were created or analyzed in this study. Data sharing is not applicable to this article.

## Supplementary Materials

The following supporting information can be downloaded at: https://www.mdpi.com/article/10.3390/jcm12103407/s1, S1: Search strategy syntax; S2: Risk of Bias assessment for each of the included studies.
