# Supplementary material for: Effects of Transcranial Electrical Stimulation on Gambling and Gaming: A Systematic Review of Studies on Healthy Controls, Participants with Gambling/Gaming Disorder, and Substance Use Disorder
Source: J Clin Med. 2023 May 11;12(10):3407. doi: 10.3390/jcm12103407 (PMC10218894; doi:10.3390/jcm12103407)
Supplement: Supplementary file 1 [file jcm-12-03407-s001.zip › jcm-2317953-supplementary.pdf]

S1. Search strategy syntax

PubMed:

	**Keywords and MeSh terms for Transcranial Electrical Stimulation**
#1	"non-invasive brain stimulation"[Title/Abstract] OR "transcranial direct current stimulation"[Title/Abstract] OR "transcranial alternating current stimulation"[Title/Abstract] OR tdcs[Title/Abstract] OR tacs[Title/Abstract] OR neuromodulation[Title/Abstract] OR "transcranial electrical stimulation"[Title/Abstract] OR "Transcranial Direct Current Stimulation"[Mesh]
	**Keywords and MeSh terms for gambling and gaming behaviours**
#2	gambl*[Title/Abstract] OR "video game*"[Title/Abstract] OR gaming [Title/Abstract] OR "risk taking" [Title/Abstract] OR "decision making" [Title/Abstract] OR impulsivity[Title/Abstract] OR BART[Title/Abstract] OR "Behavior, Addictive"[Mesh] OR "Internet Addiction Disorder"[Mesh] OR "Technology Addiction"[Mesh] OR "Gambling"[Mesh]
#3	#1 AND #2 NOT ("Animals"[Mesh] NOT "Humans"[Mesh])

Scopus:

TITLE-ABS("non-invasive brain stimulation" OR "transcranial direct current stimulation" OR "transcranial alternating current stimulation" OR tdcs OR tacs OR neuromodulation OR "transcranial electrical stimulation") AND TITLE-ABS(gambl* OR "video game*" OR gaming OR "risk taking" OR "decision making" OR impulsivity OR BART) AND (EXCLUDE (EXACTKEYWORD , "Animals") OR EXCLUDE (EXACTKEYWORD , "Animal") OR EXCLUDE (EXACTKEYWORD , "Animal Experiment")) AND (LIMIT-TO (SRCTYPE , "j"))

Web of Science:

(TS=("non-invasive brain stimulation" OR "transcranial direct current stimulation" OR "transcranial alternating current stimulation" OR tdcs OR tacs OR neuromodulation OR "transcranial electrical stimulation")) AND TS=(gambl* OR "video game*" OR gaming OR "risk taking" OR "decision making" OR impulsivity OR BART)

Refined by: Document type – articles, Research areas – Psychology or Neurosciences Neurology or Psychiatry or Behavioural Sciences or Substance abuse.

Link: https://www.webofscience.com/wos/woscc/summary/112ba9dc-4a18-4375-84b3-a215760f1ce8-689f85e8/relevance/1

S2. Risk of Bias assessment for each of the included studies

Risk of bias domains

Study	D1	D2	D3	D4	D5	Overall
Gilmore et al. 2018	-	-	+	+	+	-
Guo et al. 2018	-	-	+	+	+	-
Soyata et al. 2018	+	+	+	+	+	+
Patel et al. 2022	-	+	+	-	+	-
Boggio et al. 2010a	-	-	+	X	-	X
Fecteau et al. 2007a	-	+	+	+	+	-
Boggio et al. 2010b	-	+	+	+	+	-
Weber et al. 2014	X	+	+	+	+	X
Oulett et al. 2015	-	+	+	+	+	-
Russo et al. 2017	-	+	+	+	+	-
Yaple et al. 2017	X	-	+	+	+	X
Fecteau et al. 2007b	-	X	+	-	+	X
Ye et al. 2015a	-	+	+	+	-	-
Xiong et al. 2021	-	+	+	+	-	-
Sela et al. 2021	-	+	+	+	+	-
Minati et al. 2012	-	+	+	+	-	-
Jeong et al. 2020	-	-	+	+	+	-
Ye et al. 2015b	-	+	+	+	-	-
Yang et al. 2017	-	+	+	+	-	-
Huang et al. 2017	-	+	+	+	-	-
Verveer et al. 2020	-	X	X	+	+	X
He et al. 2016.	-	+	+	+	-	-
Martinotti et al. 2019	-	+	+	+	-	-
Alizadehgoradel et al. 2020	+	-	+	+	+	-
Ye et al. 2016	-	+	+	+	-	-
Leon et al. 2020	-	+	+	+	-	-
Wang et al 2017.	X	+	+	+	-	X
Lee et al. 2021	+	+	+	+	+	+
Wischnewski et al. 2016	-	-	+	+	-	-
Wischnewski et al. 2020	-	+	+	+	-	-
Cheng et al. 2016	-	+	+	+	-	X
Wischnewski et al. 2022	X	+	+	+	-	X
Pripfl et al. 2013	-	+	+	+	+	-
Gorini et al. 2014	-	+	+	+	-	-
Dantas et al. 2021	-	+	+	+	-	-
Fecteau et al. 2014	-	+	+	+	-	-
Nejati et al. 2018	-	+	+	+	+	-
Mattavelli et al. 2022	-	+	+	+	-	-
Wu et al. 2021	+	+	+	+	+	+
Wu et al. 2020	+	+	+	+	+	+

Domains:
D1: Bias arising from the randomization process.
D2: Bias due to deviations from intended intervention.
D3: Bias due to missing outcome data.
D4: Bias in measurement of the outcome.
D5: Bias in selection of the reported result.

Judgement
X High
- Some concerns
+ Low

The image was generated using Robvis visualization tool
